# Notes and News

**Published:** 1902-10-25

**Authors:** 


### Notes and News.

Glasgow is now entirely free from small-pox.

Arrangements have now been made to receive applications for lodgings in Madrid for the International Medical Congress in April. The booking fee or deposit is £2, to be sent with the application to the honorary secretaries, Mr. D'Arcy Power and Dr. P. Horton Smith.

The experience of Cardiff in respect to foreign lunatics gives another example of the cost of the alien to this country. During the past five years Cardiff has had to support 96 foreign lunatics at a cost of £1,586. Inquiry shows that other ports have had to bear heavy costs in the same direction.

The findings of the Commission of Inquiry into the alleged overcrowding on the *Drayton Grange* troopship show that the evils of the crowded state of the ship arose from the special circumstances of the case, which were the existence of measles on board and the want of discipline amongst the troops.

New regulations have been formulated as to the duties of medical officers in the Navy. These provide that the Inspector-General or Deputy Inspector-General in charge of a hospital in a home port is to assist the Commander-in-Chief, as required, on questions of sanitation and other matters pertaining to the medical profession and duties within the command, but he is not to leave the port without Admiralty authority first obtained. He is to inspect the marine infirmaries, dockyards, surgeries, and sick quarters of the various establishments at the port once a quarter, reporting to the Commander-in-Chief on their sanitary condition, the state of the surgical instruments, the cooking of the food for the sick, and any other special matters which may come under notice, and when required by the Commander-in-Chief he will visit ships in harbour, and similarly report on the above matters.

London has been free during one month from any death from small-pox.

The mortality from plague in India has been rising rapidly during the last few weeks.

It is proposed to form an English committee to raise a memorial to the late Professor Virchow.

Dr. Logan Taylor has been sent to the Gold Coast by the Liverpool School of Tropical Medicine to study the prevalence of ill-health on this coast. Dr. Taylor has already been to Sierra Leone, where he gained much experience in the investigation of malaria.

A new hospital for infectious diseases has been opened at Workington by the Corporation. The interior of the old hospital has been entirely removed, so that the institution is a practically new structure. On the ground floor and first floor are two large wards, containing in all 20 beds. For erection of the hospital the Corporation borrowed £5,505 from the Local Government Board.

Dr. Patrick Manson's observations on the outbreak of beriberi in the Boer camp at St. Helena are exceedingly interesting, more especially as they show that the causation of the disease is still most uncertain. In the instance of the Boer camp the most popular explanations failed to apply, the only definite feature being the marked improvement shown in the cases on removal from the spot where the disease first showed itself.

The annual sale on October 17th of the articles made solely by the patients of the Portsmouth Parish Infirmary, brought another successful year to a close for the untiring workers of the Brabazon Society. The sale was opened by the founder, the Countess of Meath, and in her remarks, after giving a short history of the origin of the scheme, she spoke highly of the quality of the work displayed. In seconding a vote of thanks to her ladyship, the medical superintendent, Dr. Chas. Knox, emphasised the point that during the many years Brabazon work had been done in the infirmary, no friction whatever had arisen, greatly owing, he felt, to the tact the ladies had shown in their visits. He was sure recreation was a good help to convalescence, and the work was a pleasure, not a task; a reward for good behaviour and willingness to help in other ways. The sale, which was well attended, provides money for presents for the patients at Christmas, as well as the necessary funds for materials used during the ensuing year. Greatly to the delight of the individual Brabazon workers, the Countess of Meath, immediately after having opened the annual sale of work, paid an unofficial visit to the Portsmouth parish infirmary, and spoke to each patient. It was an event in the lives of some of the people to actually see and talk to the founder of the scheme that they had belonged to so many years, and they highly appreciated the gracious act. The Countess visited all the infirmary, and was greatly struck by the progress, so marked, in the different stages of the buildings; the old infirmary, a building much improved, though cramped; the imbecile blocks built much later; and the present new infirmary, leaving little that could be desired.

